# Dietary intake influences gut microbiota development of healthy Australian children from the age of one to two years

**DOI:** 10.1038/s41598-019-48658-4

**Published:** 2019-08-28

**Authors:** Misa Matsuyama, Mark Morrison, Kim-Anh Lê Cao, Solange Pruilh, Peter S. W. Davies, Clare Wall, Amy Lovell, Rebecca J. Hill

**Affiliations:** 1 Children’s Nutrition Research Centre, Child Health Research Centre, Faculty of Medicine, The University of Queensland, Centre for Child Health Research, L6 62 Graham Street, South Brisbane, Queensland 4101 Australia; 2 Diamantina Institute, Faculty of Medicine, The University of Queensland, Translational Research Institute, 37 Kent Street, Woolloongabba, Queensland 4102 Australia; 3 School of Mathematics and Statistics, Melbourne Integrative Genomics, The University of Melbourne, Parkville, Victoria 3010 Australia; 4 Genie Mathématiques et Modélisation, National Institute for Applied Sciences, INSA, 135 Avenue de Rangueil, 31400 Toulouse, France; 5 Discipline of Nutrition and Dietetics, Faculty of Medical and Health Sciences, University of Auckland, 85 Park Rd, Grafton, Auckland, 1023 New Zealand

**Keywords:** Microbiology, Health sciences

## Abstract

Early life nutrition is a vital determinant of an individual’s life-long health and also directly influences the ecological and functional development of the gut microbiota. However, there are limited longitudinal studies examining the effect of diet on the gut microbiota development in early childhood. Here, up to seven stool samples were collected from each of 48 healthy children during their second year of life, and microbiota dynamics were assessed using 16S rRNA gene amplicon sequencing. Children’s dietary information was also collected during the same period using a validated food frequency questionnaire designed for this age group, over five time points. We observed significant changes in gut microbiota community, concordant with changes in the children’s dietary pattern over the 12-month period. In particular, we found differential effects on specific Firmicutes-affiliated lineages in response to frequent intake of either processed or unprocessed foods. Additionally, the consumption of fortified milk supplemented with a *Bifidobacterium* probiotic and prebiotics (synbiotics) further increased the presence of *Bifidobacterium* spp., highlighting the potential use of synbiotics to prolong and sustain changes in these lineages and shaping the gut microbiota community in young children.

## Introduction

The gut microbiota co-evolved with the human host to develop a mutual symbiotic relationship^1^. The relationship between the host and resident microbes is vital for human development and health^2^. Gut microbiota colonisation and development takes place in early life and influences short and long-term health outcomes. Such outcomes include but are not limited to the development of overweight and obesity^3–6^, allergic diseases^7–11^ and neurological disorders^12,13^. Emerging evidence suggests that the microbial community is not yet mature in adolescents^14^, therefore, it is possible that gut microbiota development continues alongside human physiological development^15^. Thus, optimising early life conditions conducive to the development of symbiosis between the host and microbiota is important.

Diet is one of the most important factors that directly affects both the composition and metabolism of the gut microbiota^16–18^, principally through their colonisation and persistence^19^. For example, dietary diversity increases available substrates for the gut microbiota, thereby, increasing microbial diversity^20^, which has been linked to health status^21^. However, the increased reliance on processed foods in the last half-century, along with antibiotic use, and shifts in lifestyle and environment has challenged the symbiotic relationship established with the resident microbes^22^ and coincides with the apparent decrease in gut microbiota diversity in Western countries^23^.

The majority of research on the effect of early life nutrition on gut microbiota development has predominantly focused on breastfeeding and/or introduction of solid food during the first year^24–34^. However, important nutritional developments and dietary changes also occur in the second year^35,36^ when children transition from a predominantly milk-based diet to table foods. Given the paucity of longitudinal research on the effect of dietary intake on gut microbiota development in early life^37^, we aimed to longitudinally examine the effect of diet on the gut microbiota of children throughout their second year of life.

## Results

### Study subjects

The gut microbiota profiles were obtained from children in the Child Health and Resident Microbes (CHaRM) study which was run in adjunct to the Growing Up Milk ‘Lite’ (GUMLi) trial. The GUMLi trial was a double blind randomised controlled trial to investigate effects of toddler milk compared to unfortified cow’s milk in healthy (i.e. free of any known disease) children from the age of one to two years. GUMLi is a fortified milk supplemented with synbiotic; *Bifidobacterium breve* M-16V, long-chain galactooligosaccharides (GOS) and short-chain fructooligosaccharides (FOS). Of 52 children enrolled in the GUMLi trial in Brisbane, 51 consented to participate in the longitudinal CHaRM study, and 48 children (94%) completed the study. Table 1 outlines the characteristics of the CHaRM study subjects and samples collected. Among the CHaRM study subjects, there were no baseline differences between the trial milk groups (GUMLi vs control) for gender, birth order, gestation, mode of delivery, duration of any breastfeeding, current breastfeeding status, antibiotic exposure, daycare attendance, pet ownership or exposure to farm animals. The GUMLi group was, however, exclusively breastfed (i.e. received breastmilk only) longer (median 19.5 weeks, range 13.0–26.0) compared with the control group (median 15.2 weeks, range 1.5–20.6) (*p* = 0.051).

**Table 1:** Summary of information for the CHaRM study subjects characteristics.

Details	n (%)
Total number of children enrolled in the longitudinal CHaRM study	51
Female subjects enrolled in the study	29 (56.8%)
Number of children withdrawn	3 (5.8%)
Final number of children completing the CHaRM study	48 (94.1%)
Female subjects completing the study	27 (56.3%)
Median duration of exclusive breastfeeding	17.3 weeks (6.5–26.0)
Median duration of any breastfeeding before the age of 2 years	41.1 weeks (20.6–65.0)
Number of children exposed to antibiotics before the age of 2 years	40 (78.4%)
Number of children completed the study who received GUMLi	24 (50.0%)

### Gut microbiota characteristics

In total, 347 gut microbiota samples were collected, of which 345 were included for analyses. After quality scoring and filtering, the entire dataset was comprised of 126 different operational taxonomic units (OTUs) and 24 identified genera.

### Shifts in gut microbiota from the age of one to two years

**Figure 1:**
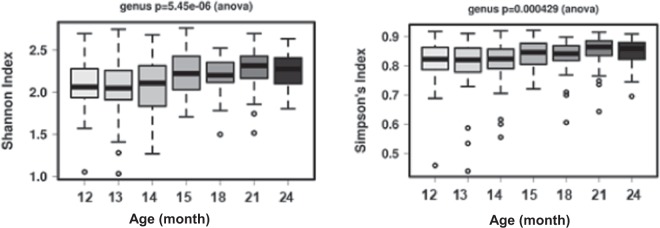
Change in microbial community number (richness) and distribution (evenness) from baseline (12 months of age) to end of study (24 months of age).

**Figure 2:**
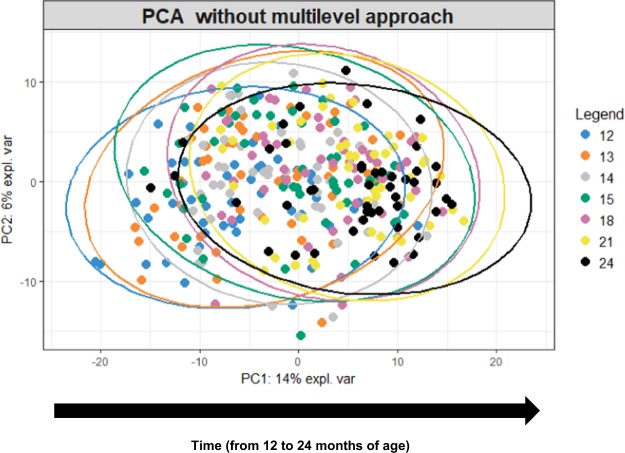
(a) Principal Component Analysis of all gut microbial OTUs collected over the 12 month period from baseline to end of study showing a gradual shift of the gut microbial community from the age of 12 to 24 months.

Alpha diversity scores significantly increased during the second year of life (*p* < 0.01). The overall richness and evenness of the microbial community expanded during this period irrespective of diet (Fig. 1). The Principal Components Analysis (PCA) highlighted a small but gradual shift of the gut microbial community with age (Fig. 2). We also observed significant changes (i.e. increase or decrease) in bacterial taxa from baseline (one year of age) to the end of study (two years of age) at phylum, family, genus and OTU levels as presented in Table S1. At the genus level, *Eubacterium*, *Veillonella*, *Oscillospira*, *Streptococcus*, *Eggerthella* and *Akkermansia* all significantly decreased during the second year of life (FDR < 0.05), while the relative abundance of *Faecalibacterium* increased during the same period. At the OTU level, the majority of those that significantly decreased in their relative abundance were assigned to the genera listed above, as well as *Lachnospiraceae* family. However, unspecified family members belonging to *Lachnospiraceae* and *Erysipelotrichaceae* and unspecified *Blautia* genus increased during this period.

### Shift in dietary pattern from the age of one to two years

The dietary patterns of children were initially explored with PCA^38^. First, analysis of all children enrolled in the GUMLi trial (n = 160) was performed to obtain robust results^39^ and clusters of food groups were visualised and identified with correlation circle plots (Fig. S1a). On the first component (explaining 16% of total variance), a shift in children’s dietary pattern was observed across time from one to two years of age (Fig. S1b). The most contributing factors to this shift is change from an “infant-like” diet represented by ‘baby’ foods at the first collection time point (yellow circle), to an “adult-like” diet by the last data collection time point. Furthermore, the analysis showed that the “adult-like” diet could be further subdivided in the second component to an ‘unhealthy’ diet represented by processed meat, savoury snacks, hot-chips/French fries and cakes (red circle); or a ‘healthy’ diet represented by meat/fish, fruit, vegetables, eggs/beans and bread/pasta (blue circle). We ran sub-group analysis of the CHaRM study participants only and found no difference in the dietary patterns.

### Shift in gut microbiota with dietary pattern from the age of one to two years

**Figure 3:**
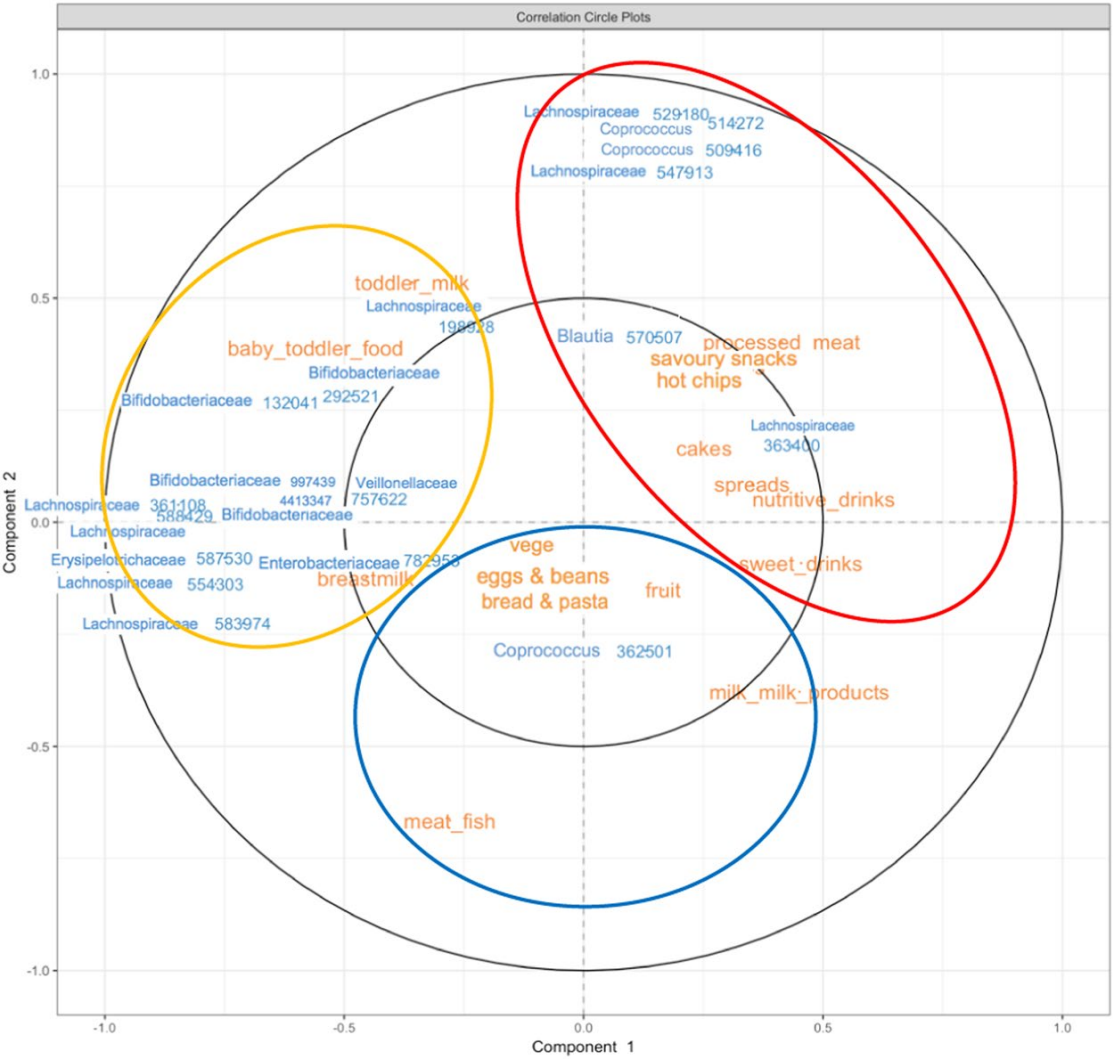
Correlation circle plot showing global analysis of gut microbial OTUs and food groups. Ellipses showing clusters of food groups correlated with OTUs. Yellow circle = ‘baby’ foods, red circle = ‘unhealthy’ foods, and blue circle = ‘healthy’ foods.

Data integration of food groups representing children’s dietary pattern with microbial OTUs from time points 0, 3, 6, 9, and 12 months was performed with sparse Partial Least Square (sPLS) analysis^40^, as shown in Fig. 3. The infant-like diet was correlated with unspecified genera belonging to *Bifidobacterium* and *Ruminococcus* as well as unknown members of *Erysipelotrichaceae* and *Lachnospiraceae* families (yellow circle). The ‘unhealthy’ adult-like diet correlated with unspecified members of *Lachnospiraceae* family and a *Coprococcus* genus (red circle). The ‘healthy’ adult-like diet correlated with a different unspecified *Coprococcus* genus (blue circle).

### Effect of children’s diet on their gut microbiota

In order to detect dynamics between specific microbial communities and food intake over the 12 months of the study period, we conducted sPLS analyses^41^ at each individual time point. The correlation between sPLS selected microbial OTUs and food groups are visualised as clustered image maps in Fig. S2. A summary of microbial taxa correlated with food groups over different time points is available in Table 2. Briefly, unspecified family members of *Lachnospiraceae*, a *Ruminococcus* genus, and a *Bacteroides* genus were positively associated with unprocessed foods (e.g. meat/fish, fruit) but negatively with processed food groups (e.g. processed meat, savoury snacks). Whereas, unspecified *Lachnospiraceae* family as well as *Blautia* and *Clostridium* genera were positively associated with processed food and negatively associated with unprocessed food groups. Unspecified *Bifidobacterium* genera were positively associated with GUMLi intake but negatively with other milk/milk products.

**Table 2:** Summary of bacterial taxa associated with food groups over different time points.

Bacteria taxa (OTU ID)	Associated food group(s)	Association	Time point(s)
*Lachnospiraceae* (363400)	Processed meat	Positive	0, 3, 6
	Hot chips (French fries)	Positive	3, 6
	Sweet drinks	Positive	3, 6
	Savoury snacks	Positive	3, 6
	Meat/fish	Negative	0, 3
*Lachnospiraceae* (554303)	Eggs/beans	Positive	9, 12
	Fruit	Positive	9
	Savoury snacks	Negative	6
	Hot chips (French fries)	Negative	6
	Nutritive drinks	Negative	6
	Sweet drinks	Negative	6
*Lachnospiraceae* (588429)	Meat/fish	Positive	3
	Eggs/beans	Positive	9, 12
	Fruit	Positive	9, 12
	Processed meat	Negative	3
*Blautia* (546876)	Savoury snacks	Positive	3
	Milk	Positive	3
	Processed meat	Positive	3
	Meat/fish	Negative	3
	Eggs/beans	Negative	12
*Ruminococcus* (583398)	Meat/Fish	Positive	3
	Processed meat	Negative	3
	Savoury snacks	Negative	6
	Nutritive drinks	Negative	6
*Coprococcus* (362501)	Milk/milk products	Positive	3, 6, 9
	Toddler milk	Negative	6, 9
*Bacteroides* (305946)	Fruit	Positive	9
	Eggs/beans	Positive	9,12
	Meat/fish	Positive	9
	Hot chips (French fries)	Negative	6
	Nutritive drinks	Negative	6
*Clostridium* (317135)	Processed meat	Positive	0
	Fruit	Positive	0
	Hot chips (French fries)	Positive	6
	Nutritive drinks	Positive	6
	Sweet drinks	Positive	6
	Savoury snacks	Positive	6
*Bifidobacterium* (292521)	Breast milk	Positive	0
	Nutritive drinks	Positive	0
	Toddler milk	Positive	6, 9,12
	Baby/toddler food	Positive	9,12
	Fruit	Negative	0
	Vegetables	Negative	0
	Milk/milk products	Negative	6, 9, 12
*Bifidobacterium* (132041)	Toddler milk	Positive	6, 9, 12
	Baby/toddler food	Positive	9, 12
	Milk/milk products	Negative	6, 9, 12

### Effect of synbiotic supplemented trial milk (GUMLi) on gut microbial community

**Figure 4:**
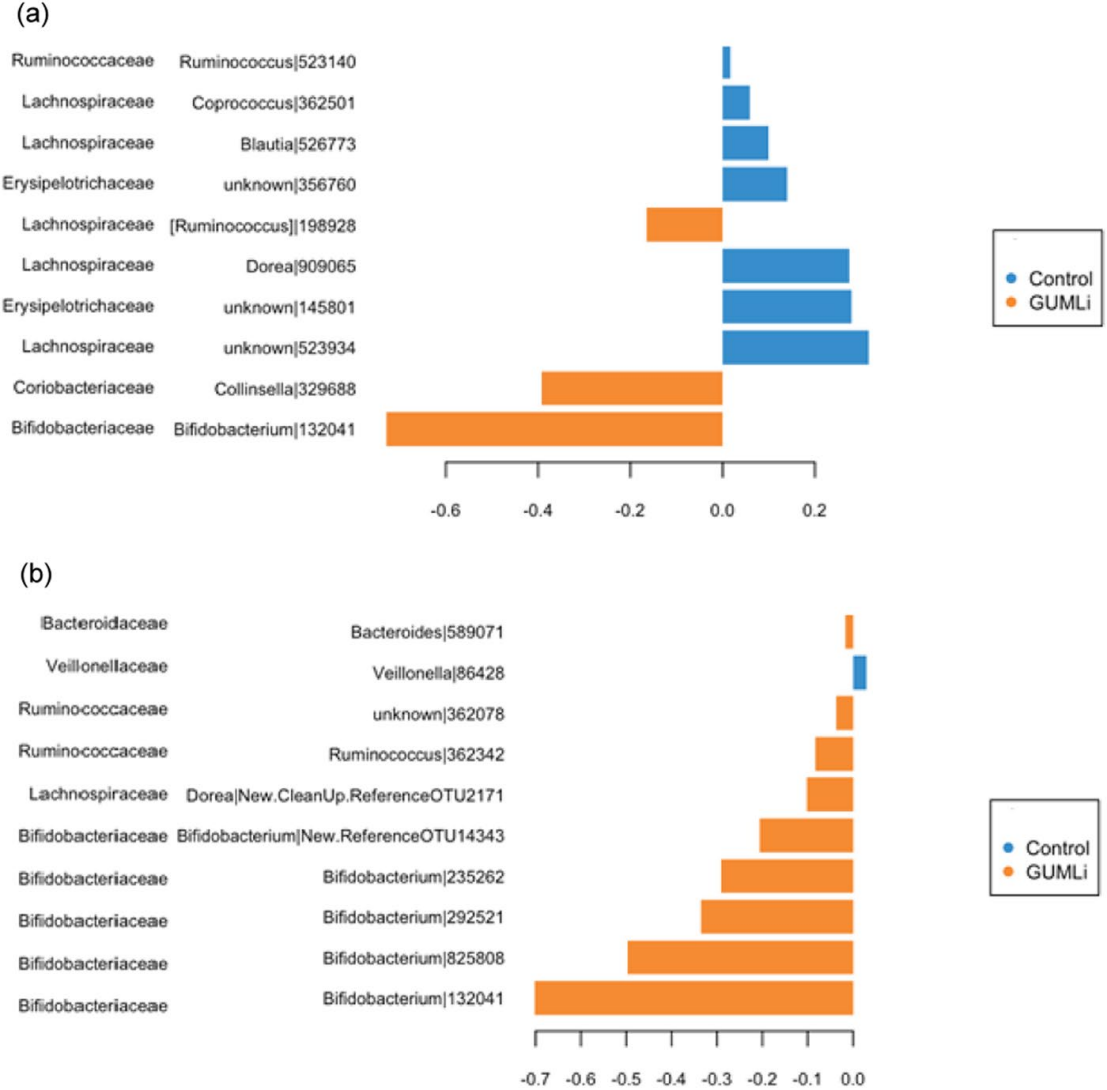
(**a**) sPLS-DA analysis of gut microbial community at month 3 of GUMLi trial and (**b**) at month 12 of GUMLi trial. The barplot highlight the most important OTUs (from bottom to top) selected by sPLS-DA, with colors indicating a maximum median abundance in a particular group.

We performed sPLS-discriminant analysis (sPLS-DA)^42^ to identify the most discriminating microbial community between the trial milk groups. Three months after initiating the trial milk, the most discriminating OTUs between the milk groups were unspecified genus belonging to *Bifidobacterium* and *Collinsella* (Fig. 4a). At month 6 of the study, another *Bifidobacterium* genus discriminated the GUMLi group, which continued until month 9 of the study (data not shown). By the end of the study (month 12), five *Bifidobacterium* genera most discriminated the microbial community between the trial milk groups and these were associated with the GUMLi group (Fig. 4b).

Linear mixed models (LMM) were used to analyse the effect of different covariates on the *Bifidobacterium* community only, as the GUMLi was supplemented with a probiotic *Bifidobacterium breve* (*B. breve*) M-16V and FOS/GOS prebiotics (Table S2). Age most influenced the shift in the *Bifidobacterium* community. However, the strongest influence was the GUMLi intake, where OTU132041 increased the most but this OTU decreased with age. Breastfeeding duration had no impact on the *Bifidobacterium* community.

### Phylogenetic analysis

**Figure 5:**
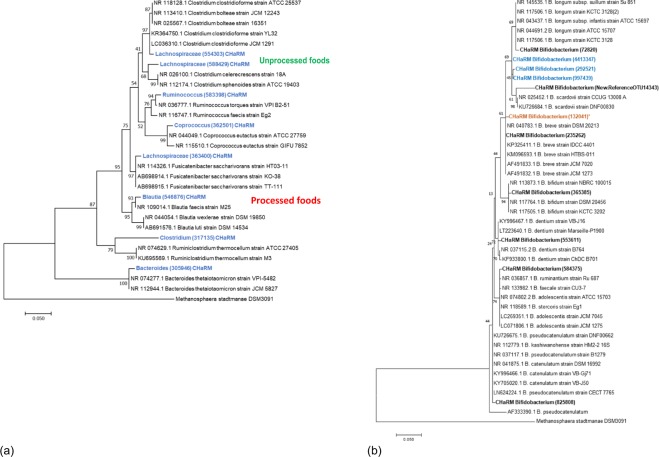
(**a**) A maximum likelihood phylogenetic tree of the 16S rRNA gene sequence of the OTUs associated with food groups over different time points. OTUs associated with processed and unprocessed food groups are labelled for Firmictues phylum. The scale bar represents 5% sequence divergence with 1000 boot straps. *Methanospheara stadtmanae* DSM3091 was used as an outgroup. (**b**) A maximum likelihood phylogenetic tree of 11 Bifidobacterium OTUs found in the CHaRM study subjects. The scale bar represents 5% sequence divergence with 1000 bootstraps. Methanospheara stadtmanae DSM3091 was used as an outgroup. An OTU associated with GUMLi closely aligned with B. breve (orange, asterisc). A cluster is formed among OTUs associated with breastfeeding at baseline (blue).

Next, we conducted phylogenetic analysis of bacterial taxa that were associated with processed or unprocessed food groups over different time points (Fig. 5a). Phylogenetic clusters were identified predominantly for Firmicutes but also a Bacteroidetes. For the Firmicutes phylum associated with unprocessed food groups, the first *Lachnospiraceae* was closely related to *Clostridium clostridioforme* or *Clostridium bolteae*, and another *Lachnospiraceae* with *Clostridium celerecrescens* and *Clostridium sphenoides*. The *Ruminococcus* was closely aligned with *Ruminococcus torques* and *Ruminococcus faecis*. The *Bacteroides* associated with unprocessed food was closely related to *Bacteroides thetaiotaomicron*. Among the OTUs associated with processed foods, the *Lachnospiraceae* aligned with *Fusicatenibacter saccharivorans*, the *Blautia* aligned with *Blautia faecis*, and the *Clostridium* aligned with *Ruminoclostridium thermocellum*. The *Coprococcus* closely aligned with *Coprococcus eutactus*, and this genus was positively associated with milk/milk product intake.

A separate phylogenetic analysis of *Bifidobacterium* was conducted (Fig. 5b). Our phylogenetic analysis is comparable to the comparative genomics study of *Bifidobacterium*^43^. *Bifidobacterium* genera that were associated with the GUMLi group were related to all *B. breve* strains used in the phylogenetic analysis. However, because *B. breve* M-16V 16S rRNA sequence has not been released to the public, we were not able to determine if these *B. breve* detected in the CHaRM cohort were a *B. breve* M-16V strain. Other *Bifidobacterium* genera grouped with *B. longum* and *B. scardovii*. Another *Bifidobacterium* genus grouped with several species, including *B. catenulatum*, *B. kashiwanohense* and *B. pseudocatenulatum*. *Bifidobacterium* genera that positively correlated with frequent breast milk intake at baseline formed a cluster in a phylogenetic analysis.

## Discussion

This study is the first to examine how diet during a child’s second year of life affects the gut microbiota. We observed correlations between dietary pattern and the bacterial community. Children’s dietary patterns shifted across time from infant-like to adult-like diet, regardless of children being involved in a trial to investigate the effect of fortified milk. The adult-like diet further deviated to either ‘healthy’ diet characterised by meat/fish, fruit, vegetables, eggs/beans and bread/pasta, or ‘unhealthy’ diet characterised by processed meat, savoury snacks, hot-chips/French fries and cakes. Such dietary patterns in young children are in line with findings from previous studies on diet of similar aged children^44,45^. We did not observe strong correlation between bacterial OTUs and breastfeeding except at the baseline. This is likely due to a considerable drop in breastfeeding rates from the age of one to two years, which is generally observed during the second year of life. These findings also suggest that the effects of diet in the second year of life have a stronger selective pressure on the gut microbiota than breastfeeding.

The gut microbiota shifted over time, demonstrated by changes in gut microbial community members and increased α-diversity indices. We observed increases in relative abundances of *Lachnospiraceae* and *Ruminococcaceae* taxa. Previous studies identified that *Lachnospiraceae* was a marker of microbiota maturation from infant-like to an adult-like community occurring in the second year of life^34,46,47^. Laursen and colleagues^34^ reported that introduction of family foods shifted the gut microbiota composition and α-diversity in nine-month-old children, suggesting increased intake of nutritionally diverse foods rich in fibre and proteins may be the main driver of gut microbial α-diversity development. We also observed that *Faecalibacterium* was the only genus, which significantly increased from the age of one to two years. *F. prausnitzii*, currently the sole species identified in the *Faecalibacterium* genus^48^ was previously reported as one of the indicators of gut microbiota maturity in young children^46,47^. They are important butyrate producers and have anti-inflammatory potential^49^ and *F. prausnitzii* is often the most abundant gut bacterium found in healthy adults^50^. An increase in *Faecalibacterium* abundance with age in this otherwise healthy CHaRM study cohort further suggests *F. prausnitzii* as one of the markers for gut microbiota maturity during the developmental period.

We observed trends in association between gut microbiota with food groups, particularly with members of the Firmicutes phylum. An unspecified member of *Lachnospiracea* family has shown persistent positive correlations with processed foods and negative correlations with unprocessed foods over several study time points (0, 3, 6 month of study). A phylogenetic analysis showed that this OTU closely aligned with *Fusicatenibacter saccharivorans*, a novel species of the *Lachnospiraceae* family isolated from human faeces^51^. Processed food is an epitome of a modern ‘Western’ diet. The increased consumption of processed foods equates to increased exposure to food additives^52^, and a number of studies have identified the negative effect of food additives on gut microbiota^53–55^. In contrast, some OTUs positively correlated with unprocessed foods, but negatively with processed food, suggesting that these bacteria may have a capacity for better growth in the presence of protein rich foods, rather than processed carbohydrate/sugar rich foods. Another unspecified *Lachnospiraceae* closely aligned with *C. bolteae* and *C. clostridioforme* in the phylogenetic analysis. Higher abundance of *C. bolteae* has been remarked upon as part of cross-sectional studies of autism-spectrum disorder^56^. Whilst we did not specifically quantify this particular *Lachnospiraceae*, its relative abundance decreased over time (Table S1). Another *Lachnospiraceae* most closely aligned with *C. celecrescens* and *C. sphenoides*, were also found to decrease over time. Changes in the relative abundances of *C. sphenoides* have been observed with a decrease in cholesterol intake in obese adults^57^, suggesting their potential role as microbial biomarkers for dietary responsiveness in otherwise healthy children.

Bifidobacteria are widely used as probiotics at all ages. The CHaRM study subjects were enrolled in a randomised controlled trial investigating the effect of GUMLi, a fortified milk supplemented with a *B. breve* M-16V probiotic and GOS/FOS prebiotics. Therefore, additional analyses were carried out to specifically investigate how the *Bifidobacterium* community may be affected by diet and/or type of milk products consumed. Generally, the absolute and relative abundance of *Bifidobacterium* is greater for breastfed infants^58^, and the *Bifidobacterium* community also “matures” with age^59^. We observed decreased abundance of a certain *Bifidobacterium* genus as children aged, whereas, other *Bifidobacterium* genera increased their abundance. There were positive correlations between a few *Bifidobacterium* genera with breast milk intake (i.e. breastfeeding) and these genera clustered closely in a phylogenetic analysis (Fig. 5b) but there were no close relatives to assign these strains in our analysis. Although children in the GUMLi group had a longer exclusive-breastfeeding duration, overall, breastfeeding had no impact on *Bifidobacterium* community during the second year of life, when adjusted for other covariates. A *Bifidobacterium* genus which significantly increased with GUMLi intake was closely related to *B. breve* strains in the phylogenetic analysis, and further studies using quantitative PCR is required to determine the presence of the probiotic *B. breve* M-16V. In addition, whether the effect on *Bifidobacterium* community in the GUMLi group was specifically due to the probiotic *B. breve* M-16V, or the potential role of the GOS and FOS prebiotic mixture that may have contributed to this, requires further investigation.

The CHaRM study has made novel contributions to research in early life gut microbiota development, however, there are a number of study limitations. Further studies would benefit from the following improvements. Due to the small sample size of this study, there was insufficient statistical power to define significance at the OTU level of classification in the longitudinal modelling. However, our observation of increased abundance of bacterial families, including *Lachnospiraceae* and *Ruminococcaceae*, as well as the genus *Faecalibacterium* are in line with previously reported gut microbiota profiles of similar aged children. For the dietary assessment, this study used a FFQ, designed to reflect long-term dietary patterns. Ideally, detailed dietary-records over several days are better to assess the effect of diet on gut microbiota^37^, but the respondent burden is larger and time consuming^60^. Researchers must choose a balance between participant (parents) burden and the data collected^61^ and while parents and caregivers in this 12-month study did comply with the FFQ over five time points, it was burdensome and could not be expanded.

Despite these constraints, our study still revealed trends in the dietary change and associated microbial OTUs, providing new insights into the influence of diet on the development of the gut microbiota during the second year of life. We believe this is the first study to assess the gut microbiota profile together with dietary information collected throughout the second year of life. It, thereby, provides a valuable time-series dataset allowing for monitoring the trends in microbial community shifts in early life along with dietary intake. A strength of this research is that we applied a range of multivariate projection-based methods (i.e. PCA, sPLS and sPLS-DA) to investigate the effect of diet on the gut microbial community, and at different time points. The analysis enabled us to capture diversity within Firmicutes phylum members showing different capacities to grow with either processed or unprocessed foods. The synbiotic-supplemented milk appears to have facilitated recruitment and expansion of *Bifidobacterium* community members among children who consumed this milk over the 12-month period.

## Materials and Methods

### Subject and sample collection and analysis

The stool samples analysed and reported here were collected from children participating in the CHaRM study conducted in Brisbane, Australia. These children were recruited in a multi-centre trial (the GUMLi trial) investigating the effect of growing up milk (i.e. fortified milk for young children) compared to unfortified cow’s milk on various outcomes in childhood.

Details of the study methodology are available in Supplementary Information. Briefly, the intervention milk (GUMLi) was a micronutrient fortified milk with reduced energy and protein content compared to other GUM available in the market, and supplemented with probiotic *B. breve* M-16V and prebiotics, long-chain GOS and short-chain FOS. The control milk was an unfortified cow’s milk and both milks were in powder form and unidentifiable. Stool samples were collected from the Brisbane GUMLi trial participants who agreed to partake the CHaRM study by their mother or caregiver and collected at 0 (baseline), 1, 2, 3, 6, 9 and 12 months into the trial.

We used the Eating Assessment in Toddlers Food Frequency Questionnaire (EAT FFQ)^38^ to assess the dietary intake of the GUMLi trial participants at 0, 3, 6, 9, and 12 months of the study. The common food groups used to explore dietary patterns are detailed in Table S3. Other information such as breastfeeding, mode of delivery and antibiotic usage, as well as other demographic and relevant data were obtained from mothers/caregivers during the GUMLi trial data collection.

### Ethics, consent and permission

Ethical approvals were obtained from the University of Queensland Human Research Ethics Committee (reference 2014001318) and the Northern B Health and Disability Ethics Committee of the Ministry of Health, New Zealand (HDEC reference number 14/NTB/152). Written informed consent was obtained from parents/guardians on the participating child’s behalf, prior to enrolment in the trial. Additional consent was obtained for the participation in the CHaRM study in Brisbane. All experiments were performed in accordance with relevant guidelines and regulations.

### Gut microbiota DNA extraction from stool samples

Gut microbiota DNA was extracted using the repeated beating and a column technique^62^ adapted for use with the automated Maxwell 16 MDx system (Promega).

Sub-samples of stool (0.15 g) were transferred into a 2 mL screw-capped tube containing 0.4 g of sterile zirconia beads (0.1 mm and 1 mm diameter). Into this tube, 600 μL of lysis buffer (500 mM NaCl, 50 mM TRIS-HCl (pH 8.0), 50 mM EDTA and 4% [w/v] sodium dodecyl sulfate) was added and homogenised in the Precellys 24 homogeniser (Bertin Corp) at 5000 rpm, for 3 × 60 second intervals. The homogenised mixture was then incubated at 70 °C for 15 minutes, with gentle shaking by hand every 5 minutes. After incubation, the mixtures were then centrifuged at 4 °C/RT for 5 minutes at 16,363 rcf. The supernatant was transferred to a fresh 1.5 ml micro centrifuge tube and 30 μL of Proteinase K was added to the supernatant and then vortexed for 30 seconds, then incubated at 56 °C for 20 minutes. The mixtures were then transferred to the well of Maxwell 16 MDx cartridges, and 65 μL of elution buffer (Promega, catalogue no. AS1290) was added to elution tubes. The non-template control (NTC) was placed as a quality control measure, for each new batch of lysis buffer and elution buffer. After the automated DNA purification was completed, purified DNA in elution buffer were placed on a magnetic stand to remove magnetic particles, and the supernatant carefully transferred to a new micro-centrifuge tube. To each sample, 2 μL of RNase (10 mg/ml) was added then incubated at 37 °C for 15 minutes. The DNA concentrations were measured using a Nano-Drop Lite Spectrophotometer (Thermo Fisher Scientific). DNA samples were then normalised to a concentration of 5 ng/μL and checked for their quality by PCR.

The PCR was carried out with a total volume of 25 uL comprised of 12.5 μL 2XMango Mix (Bioline), 9 μL H2O, 1.5 μL MgCl2 50 mM, 0.5 μL 10 μM primers 341F-CCTACGGGNGGCWGCAG and 805R-GACTACHVGGGTATCTAATCC and 1 μL of 5 ng/μL template. This primer pair was chosen for its coverage and reduced bias, based on an experimental evaluation of 512 primer pairs^63^. The thermo-cycling condition was 1 cycle of 3 minutes at 95 °C, followed by 25 cycles of 30 seconds each at 95 °C, 55 °C, 72 °C, and then 1 cycle of 5 minutes at 72 °C and hold at 4 °C. The PCR products were then analysed with 1% agarose gel electrophoresis (1% agarose + 1 × TAE buffer). Any samples with unsuccessful PCR were repeated at different concentrations (1, 5, 0.5 and 10 ng/μL) until amplification was achieved. Four DNA samples that did not amplify were cleaned using the phenyl chloroform extraction method and purified using the ethanol precipitation method. DNA samples were stored at −30 °C prior to 16S rRNA sequencing. The amplicon libraries were created from the V3/V4 hypervariable regions of the bacterial 16S rRNA gene using 341F and 805R primers before being subjected to 16S rRNA sequencing with the MiSeq platform (Illumina).

### Bioinformatics

Raw 16S rRNA sequences were joined, demultiplexed and quality controlled using the Quantitative Insights Into Microbial Ecology (QIIME) version 1.9.1 pipeline^64^. Details of scripts are available in Supplementary Information. The chimera check and removal was conducted using USEARCH version 6.1.544^65^. The OTU was aligned using PyNAST^66^ with a 97% sequence similarity threshold against the Greengenes database version 13.8^67^ using open reference picking method in QIIME. The OTUs with less than 0.1% of total sequence were filtered and samples with less than 1000 read counts were discarded. Two samples produced less than 1000 reads and one sample produced 1448 reads, with the remaining samples producing more than 2000 reads (median 12318 reads per sample). Based on these findings we chose to work with the data produced from 345 samples. The data were normalised by Total Sum Scaling (TSS) and then transformed using the Centred Log Ratio (TSS + CLR) for downstream analysis of composition data^68^ in both mixOmics^69^ and Calypso^70^. The read counts produced from the four no template controls were very low (7–41) and thereby not considered further.

### Statistical analysis

To verify differences in factors that may potentially influence gut microbiota profiles among the CHaRM study subjects, t-test was used for normally distributed or Mann-Whitney test for non-normally distributed continuous variables. Comparison between categorical variables were performed using a Chi-square test or Fisher’s exact test when appropriate. These tests were carried out in Stata (version 13.1, StataCorp).

### Gut microbiota analysis

To determine trends in α-diversity indices, we used Calypso’s Diversity Page for analysis of microbial diversity. Changes (i.e. increase or decrease) in median bacterial taxa abundance from baseline to the end of study at phylum, family, genus and OTU levels investigated using Wilcoxon sign rank test in Calypso’s ‘Stats Page’ for statistical comparison of sample groups. *P*-values were adjusted for multiple testing using false discovery rate (FDR) and FDR < 0.05 was considered as statistically significant.

We used the mixOmics R package^69^ to explore the effect of diet on gut microbial community. The gut microbial community, as well as the children’s dietary patterns over the 12-month period were initially visualised with PCA. We applied the sPLS method to explore relationships between microbial OTUs and food groups at corresponding dietary data collection (i.e. 0, 3, 6, 9, and 12 month of study). The sPLS ‘canonical mode’ was used to identify the most correlated bacterial OTU and diet and visualised with clustered image maps^40^. The sPLS-DA, an extension of sPLS, enables the selection of most discriminative variables (i.e. trial milk) to classify the samples^42^ was used to investigate the effect of trial milk on gut microbiota. Cross-validation (5 fold cross-validation repeated 50 times) was used to select the optimum number of parameters (i.e. the number of components and the number of variables to select on each component) based on classification performance.

As the trial synbiotic milk (GUMLi) contained a *Bifidobacterium* probiotic, we fitted Linear Mixed Model (LMM)^71^ using the R package ‘nlme’^72^ to analyse the effect of GUMLi and other covariates on *Bifidobacterium* OTUs. The following covariates were chosen based on the relevance with the gut microbial community: age, trial milk group, duration of any breastfeeding, antibiotic exposure since birth, and dietary pattern. Previously, we identified that breastfeeding was the most significant factor that altered the gut microbial community at one year of age (*p* < 0.05)^73^. Breastfeeding status indicated by duration (in weeks) fitted this modelling better, rather than the breastfeeding status (i.e. yes or no) at each time point. We used dietary pattern scores to analyse the overall effect of diet on the microbial community. Dietary pattern 1 represents a shift from baby-like to adult-like diet, whereas, dietary pattern 2 represents a shift from unhealthy to healthy diet. *P*-values were adjusted for multiple testing using Benjamini Hochberg FDR^74^, and FDR < 0.05 was considered statistically significant.

### Phylogenetic analysis

Sequences related to OTUs associated with food groups over different time points were run through blastn standard nucleotide BLAST^75^ to identify closely related organisms. The 16S rRNA sequences of these organisms were retrieved from NCBI and used for phylogenetic reconstruction. These were aligned with Arb SILVA (https://www.arb-silva.de/) and imported into MEGA7^76^ for phylogenetic analysis. The maximum likelihood method based on the Kimura 2-parameter model^77^ was used to infer the evolutionary tree, evaluated with 1000 bootstrap replications.

## Supplementary information

Dietary intake influences gut microbiota development of healthy Australian children from the age of one to two years - supplementary information

## Data Availability

The datasets generated during and/or analysed during the current study are available from the corresponding author on reasonable request.
