# Supplementary material for: Dietary intake influences gut microbiota development of healthy Australian children from the age of one to two years
Source: Sci Rep. 2019 Aug 28;9:12476. doi: 10.1038/s41598-019-48658-4 (PMC6713781; doi:10.1038/s41598-019-48658-4)
Supplement: Supplementary file 1 — Dietary intake influences gut microbiota development of healthy Australian children from the age of one to two years - supplementary information [file 41598_2019_48658_MOESM1_ESM.pdf]

Dietary intake influences gut microbiota development of healthy Australian children from the age of one to two years

Misa Matsuyama, Mark Morrison, Kim-Anh Lê Cao, Solange Pruilh, Peter SW Davies, Clare Wall, Amy Lovell and Rebecca J Hill

Supplementary Information

Table S1 Median bacterial taxa abundance at 12 and 24 months of age (increase / decrease, bold FDR<0.05)

Phylum	12 m	24 m	P-value (FDR)
**Firmicutes**	2.49	5.13	0.000033 (0.00016)
**Actinobacteria**	1.38	3.13	0.0006 (0.0015)
**Bacteroidetes**	0.85	2.75	0.00088 (0.0015)

Family	12 m	24 m	P-value (FDR)
Unclassified Clostridiales	1.75	3.92	0.000038 (0.00065)
Ruminococcaceae	4.78	6.31	0.0048 (0.041)
Clostridiaceae	3.22	4.58	0.046 (0.26)

Genus	12 m	24 m	P-value (FDR)
Eubacterium	5.22	1.36	3.1E-07 (5.9E-06)
Veillonella	4.89	1.44	3.7E-07 (5.9E-06)
Oscillospira	4.2	1.2	0.000016 (0.00017)
Faecalibacterium	1.41	4.22	0.000035 (0.00028)
Streptococcus	5.54	4.5	0.00029 (0.0019)
Eggerthella	4.25	2.31	0.00082 (0.0044)
Akkermansia	4.76	1.85	0.0085 (0.039)
Ruminococcus	7.04	6.06	0.013 (0.052)
Unclassified *Clostridiales*	3.95	5.49	0.015 (0.053)
Unclassified *Lachnospiraceae*	7.31	7.07	0.033 (0.089)
Bifidobacterium	7.97	7.35	0.035 (0.089)
Bacteroides	7.75	6.8	0.035 (0.089)
Coprococcus	4.23	5.33	0.036 (0.089)

OTU	12 m	24 m	P-value (FDR)
***Erysipelotrichaceae* (356760)**	9.84	3.51	2.2E-10 (2.8E-08)
***Eubacterium dolichum* (587530)**	8.58	3.77	1.4E-09 (8.8E-08)
***Enterobacteriaceae* (782953)**	9.67	4.14	3.1E-09 (1.3E-07)
***Veillonella dispar* (757622)**	9.57	4.46	5.1E-09 1.6E-07
***Lachnospiraceae* (583974)**	7.95	1.52	8.7E-09 (2.1E-07)
***Ruminococcus gnavus* (360015)**	11.23	5.54	1E-08 (2.1E-07)
***Erysipelotrichaceae* (145801)**	9.08	4.5	1.5E-08 (2.7E-07)
***Lachnospiraceae* (554303)**	8.91	3.61	4.3E-08 (6.8E-07)
***Streptococcus* (593803)**	9.64	6.5	4.9E-08 (6.9E-07)
***Streptococcus* (1082539)**	8.4	5.47	1.1E-07 (1.4E-06)
***Oscillospira* (335550)**	8.77	4.55	1.3E-07 (1.5E-06)
***Streptococcus* (579608)**	10.03	6.62	1.6E-07 (1.7E-06)
***Veillonella dispar* (86428)**	7.17	2.63	3.4E-07 (3.3E-06)
***Lachnospiraceae* (361108)**	6.61	-8.8	4E-07 (3.6E-06)
***Bifidobacterium* (132041)**	9.61	4.83	1.1E-06 (9.2E-06)
***Lachnospiraceae* (588429)**	7.64	4.28	3.2E-06 (0.000025)
***Eggerthella lenta* (1141218)**	8.72	5.04	3.4E-06 (0.000025)
***Dorea* (659361)**	9.61	5.52	0.00002 (0.00014)
***Lachnospiraceae* (84589)**	-7.2	6.48	0.000044 (0.00029)
***Ruminococcus gnavus* (84766)**	7.39	3.89	0.00035 (0.0022)
***Bacteroides fragilis* (351231)**	8.16	4.62	0.00048 (0.0029)
***Dorea* (909065)**	7.12	3.71	0.00057 (0.0033)
***Blautia* (546876)**	-7.3	5.93	0.00063 (0.0035)
***Bifidobacterium longum* (72820)**	10.85	8.55	0.00068 (0.0036)
***Clostridiaceae* (606927)**	8.98	6.8	0.00089 (0.0045)
***Erysipelotrichaceae* (580008)**	-7.2	7.6	0.0043 (0.021)
Clostridiales (470382)	-3.8	7.19	0.0084 (0.039)
Parabacteroides distasonis (585914)	5.17	4.11	0.029 (0.13)
Blautia (198532)	3.83	5.67	0.03 (0.13)
Lachnospiraceae (353149)	3.1	5.46	0.032 (0.13)
Lachnospiraceae (701221)	6.63	7.46	0.039 (0.16)
Clostridiaceae (338222)	6.87	5.58	0.046 (0.18)

Table S2 Linear Mixed Models were fitted to each *Bifidobacterium* OTU individually to study the effect of potentially influential covariates. P-values corrected for multiple testing (False Discovery Rate). Bold red indicated adjusted $p < 0.05$.

	**Age**		**GUMLi**		**Breastfeeding**		**Antibiotics**		**Diet 1**		**Diet 2**	
***Bifidobacterium* OTU**	C	SE	C	SE	C	SE	C	SE	C	SE	C	SE
825808	**0.077**	**0.031**	-0.253	0.390	-0.000	0.007	0.220	0.335	0.084	0.082	-0.035	0.076
553611	-0.009	0.023	-0.654	0.319	0.002	0.006	0.044	0.251	-0.014	0.059	0.008	0.055
4413347	-0.028	0.023	0.026	0.250	-0.001	0.005	-0.578	0.235	-0.117	0.061	0.010	0.056
72820	0.036	0.033	0.174	0.365	-0.014	0.006	0.064	0.335	-0.022	0.086	-0.018	0.079
292521	-0.010	0.021	**0.592**	**0.187**	0.001	0.004	-0.341	0.192	-0.079	0.054	0.011	0.049
235262	**0.041**	**0.015**	0.040	0.138	0.003	0.003	0.074	0.140	0.064	0.039	0.052	0.036
584375	-0.010	0.030	-0.387	0.394	-0.008	0.007	**0.951**	**0.327**	0.169	0.079	0.080	0.073
132041	**-0.116**	**0.031**	**1.182**	**0.423**	0.011	0.008	-0.134	0.338	-0.205	0.080	0.028	0.074
997439	-0.043	0.025	-0.021	0.239	0.001	0.005	-0.481	0.238	-0.126	0.064	-0.027	0.059
365385	-0.023	0.040	-0.535	0.524	0.003	0.009	-0.013	0.430	0.093	0.103	-0.151	0.096
14343	**0.080**	**0.023**	-0.226	0.214	0.003	0.004	0.293	0.216	0.145	0.061	0.040	0.056

GUMLi = GUMLi (trial milk intervention) group, Breastfeeding = duration of breastfeeding, Antibiotics = exposure to antibiotics (yes), Diet 1 = shift from a baby-like to adult-like diet, Diet 2 = shift from healthy to unhealthy diet. C=coefficient, SE= standard error, **$p < 0.05$ (adjusted)**

Table S3 EAT FFQ food groups and food items in each group for PCA analysis.

Food group	Food frequency questionnaire food item
Baby and toddler foods	Little Kids cheesy ravioli (Wattie's), other Little Kids meals (Wattie's), other toddler meals, Simply Create meat pouches (Wattie's), baby rice, baby muesli from packet, vegetable-based meals, meat-based meals, pasta- or rice-based savory meals, rice- and other cereal-based desserts, custard and other milk-based desserts, fruit purée, fruit-based desserts, junior fruit drink
Bread, pasta, rice, low-sugar cereal	White bread, buns (not iced), crumpets, wholemeal or wholegrain bread or buns, rice cakes, rice wheels, crispbreads, Weet-bix (Sanitarium Health Food Company), Fruity-bix (Sanitarium Health Food Company), porridge (not instant porridge in individual servings), cornflakes, rice bubbles, other breakfast cereals (<15 g sugar per 100 g), rice, canned spaghetti, other pasta, fruit bread, currant buns
Meat	Other chicken, other fish, mince and patties, other meat (beef, lamb, pork, mutton)
Processed meat	Chicken nuggets or shapes, fish fingers or shapes, battered or crumbed fish, sausages, saveloys (frankfurters), hot dogs (including vegetarian), ham, bacon, luncheon meats, meat pies, and sausage rolls
Eggs and beans	Eggs, canned beans (including baked beans), hummus (chickpea dip)
Vegetables	Potato and kumara (sweet potato) (boiled, baked, microwaved, mashed), frozen mixed vegetables, carrot, pumpkin, green peas, sweet corn, broccoli and cauliflower, green leafy vegetables, salad greens, raw tomato, cooked tomato (pasta sauce, canned tomato), other vegetables
Fruit	Canned fruit, banana, apples, pears, oranges, mandarins, kiwifruit, grapes, berries (fresh or frozen), dried fruit, avocado, other fruit
Milk and milk products	Low-fat cow's milk as a drink, low-fat cow's milk on cereal or other food (not custard or sauces), whole cow's milk as a drink, cow's milk on cereal or other food (not custard or sauces), other milk as a drink, cheese (including in recipes), yogurt, dairy food, white sauce, custard, and other milk puddings
Breast milk	Breastmilk
Infant and follow-up formula	Infant formula, follow-up formula
Butter and margarines	Butter, margarines
Sweet foods (cakes, cookies, puddings, confectionary, sweet snacks, sweet cereals)	Ice cream, cookies—coated (with chocolate, icing, yogurt), cookies—other, cakes, muffins, scones, croissant, sweet buns, iced buns, pastries, puddings, chocolate, lollipops, bars (muesli, nut, cereal, or puffed rice), fruit leather, fruit strings, fruit rollups, other breakfast cereals
Sweet drinks	Fruit drinks, cordial (nonalcoholic sweetened fruit drink), powdered fruit drinks, carbonated sodas (eg, lemonade [lemon-lime soda], cola)
Fries, roast potato and kumara (sweet potato)	Fries, potato shapes, roast potato or kumara (sweet potato)
Savory snacks	Crackers (wheat-, rice-, or corn-based), instant noodles, potato chips, corn chips, extruded corn snacks
Fruit or milk drinks	Fruit juice (reconstituted or freshly squeezed), flavored milk (eg, chocolate, breakfast drinks)

Reproduced from Journal of the Academy of Nutrition and Dietetics, 115 (4), Mills, V.C. *et al*., Relative validity and reproducibility of a food frequency questionnaire for identifying the dietary patterns of toddlers in New Zealand, 551-558, Copyright (2015), with permission from Elsevier

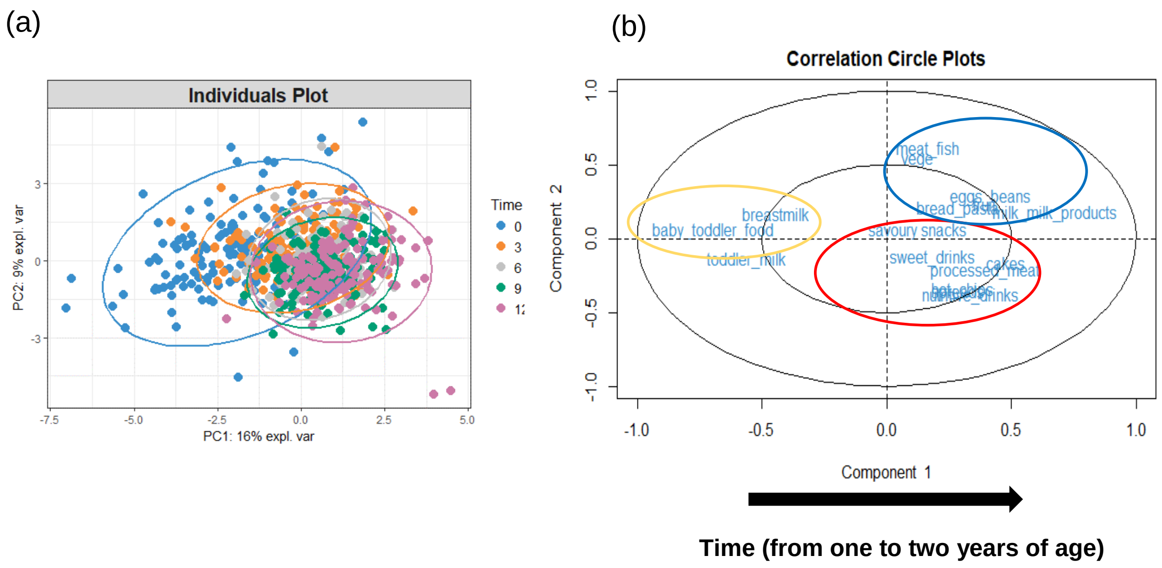

Figure S1 PCA individual plot coloured by time of collection (a), and variable correlation circle plot (b) of global dietary pattern of all GUMLi study subjects from baseline to end of study. In correlation circle plot, coloured circles show clusters of food groups, where yellow circle = ‘baby’ foods, red circle = ‘unhealthy’ foods, and blue circle = ‘healthy foods.

Figure S2 Clustered image maps show correlations between selected bacterial OTUs and selected food groups aby sPLS at each individual time points 0, 3, 6, 9, 12.

Time point 0 (baseline)

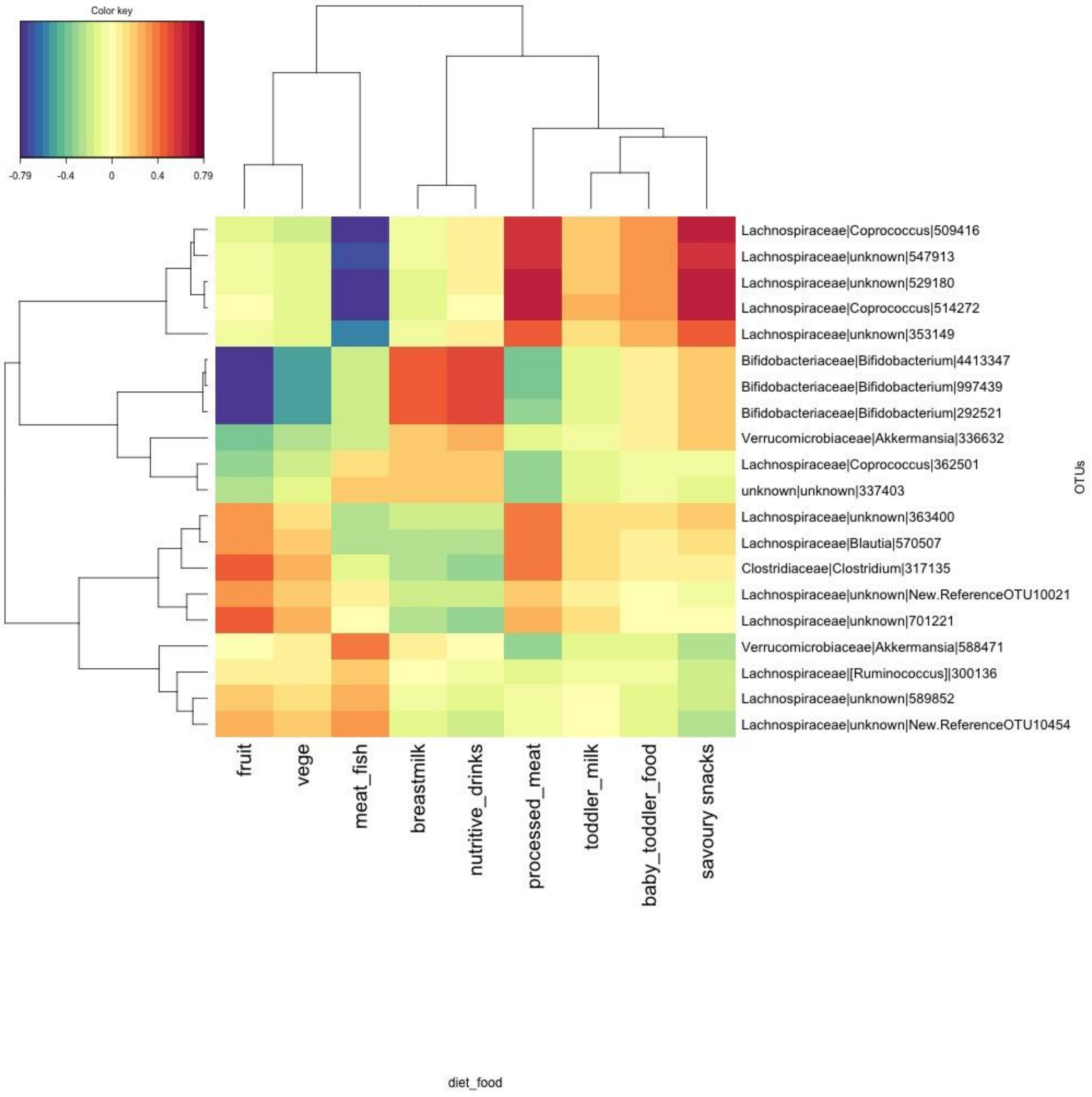

Time point 3

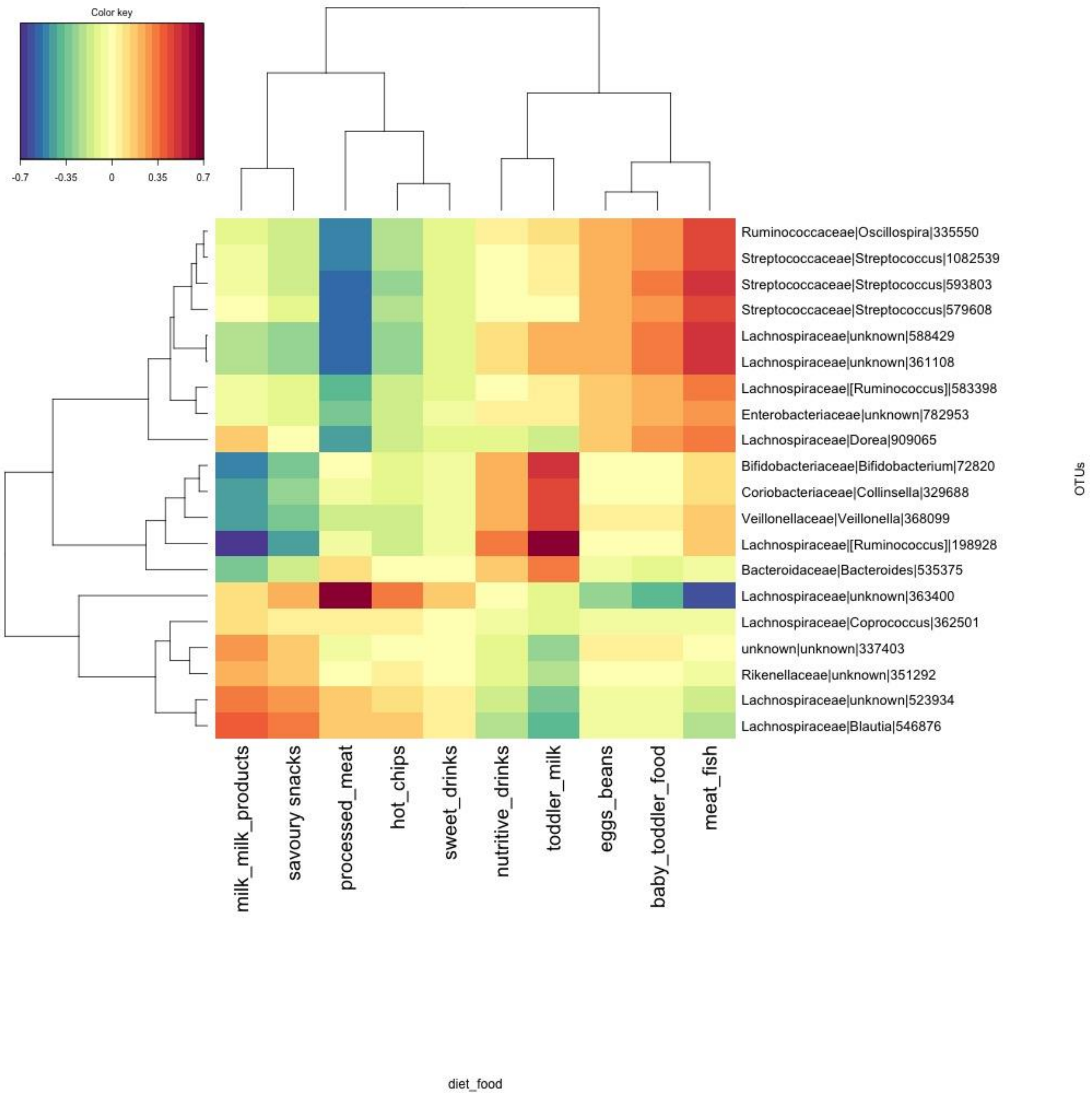
Color key
-0.7
-0.35
0
0.35
0.7
Ruminococcaceae|Oscillospira|335550
Streptococcaceae|Streptococcus|1082539
Streptococcaceae|Streptococcus|593803
Streptococcaceae|Streptococcus|579608
Lachnospiraceae|unknown|588429
Lachnospiraceae|unknown|361108
Lachnospiraceae|[Ruminococcus]|583398
Enterobacteriaceae|unknown|782953
Lachnospiraceae|Dorea|909065
Bifidobacteriaceae|Bifidobacterium|72820
Coriobacteriaceae|Collinsella|329688
Veillonellaceae|Veillonella|368099
Lachnospiraceae|[Ruminococcus]|198928
Bacteroidaceae|Bacteroides|535375
Lachnospiraceae|unknown|363400
Lachnospiraceae|Coprococcus|362501
unknown|unknown|337403
Rikenellaceae|unknown|351292
Lachnospiraceae|unknown|523934
Lachnospiraceae|Blautia|546876
OTUs
milk_milk_products
savoury snacks
processed_meat
hot_chips
sweet_drinks
nutritive_drinks
toddler_milk
eggs_beans
baby_toddler_food
meat_fish
diet_food

Time point 6

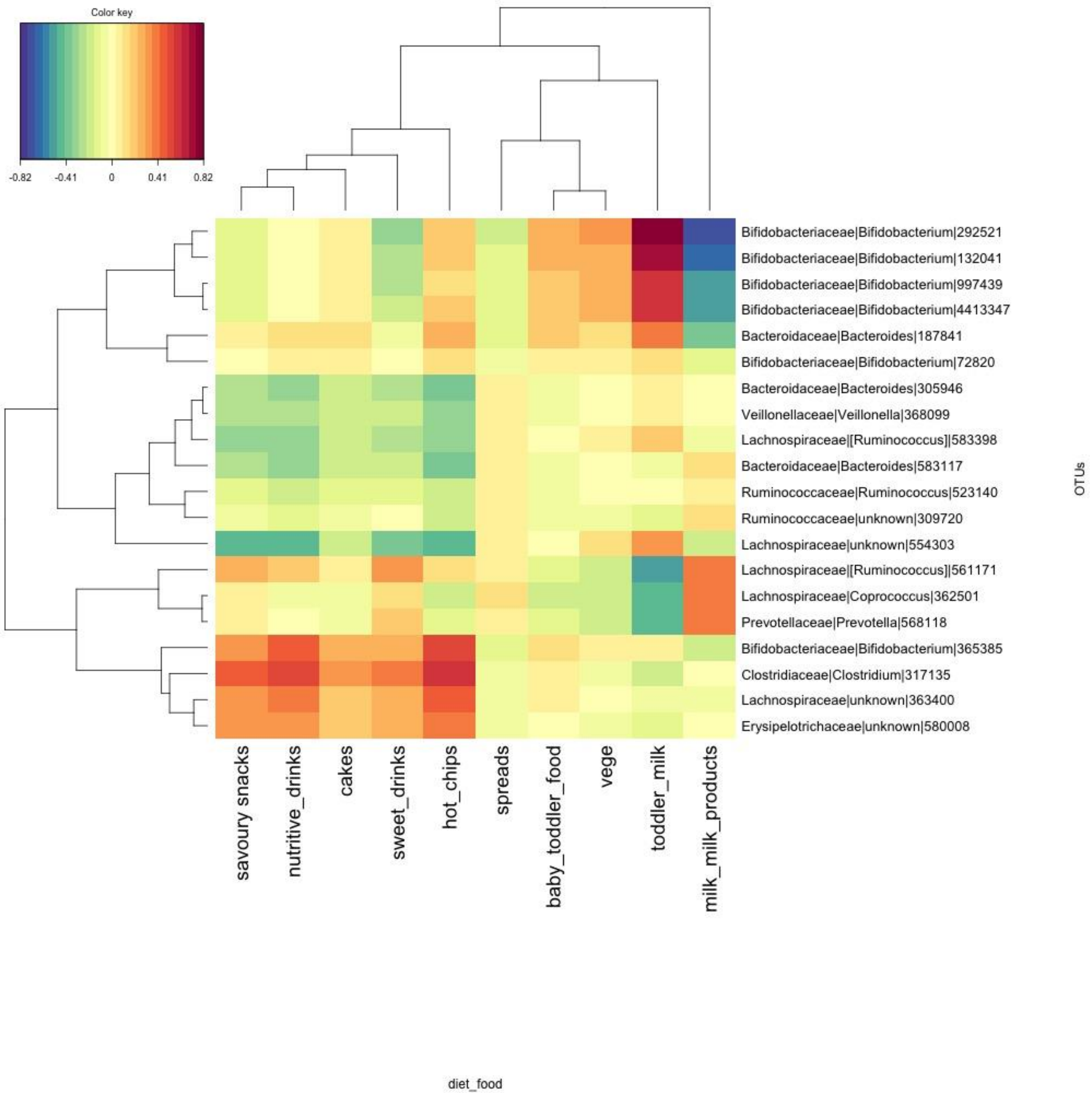

Color key
-0.82
-0.41
0
0.41
0.82
Bifidobacteriaceae|Bifidobacterium|292521
Bifidobacteriaceae|Bifidobacterium|132041
Bifidobacteriaceae|Bifidobacterium|997439
Bifidobacteriaceae|Bifidobacterium|4413347
Bacteroidaceae|Bacteroides|187841
Bifidobacteriaceae|Bifidobacterium|72820
Bacteroidaceae|Bacteroides|305946
Veillonellaceae|Veillonella|368099
Lachnospiraceae|[Ruminococcus]|583398
Bacteroidaceae|Bacteroides|583117
Ruminococcaceae|Ruminococcus|523140
Ruminococcaceae|unknown|309720
Lachnospiraceae|unknown|554303
Lachnospiraceae|[Ruminococcus]|561171
Lachnospiraceae|Coprococcus|362501
Prevotellaceae|Prevotella|568118
Bifidobacteriaceae|Bifidobacterium|365385
Clostridiaceae|Clostridium|317135
Lachnospiraceae|unknown|363400
Erysipelotrichaceae|unknown|580008
savoury snacks
nutritive_drinks
cakes
sweet_drinks
hot_chips
spreads
baby_toddler_food
vege
toddler_milk
milk_milk_products
OTUs
diet_food

Time point 9

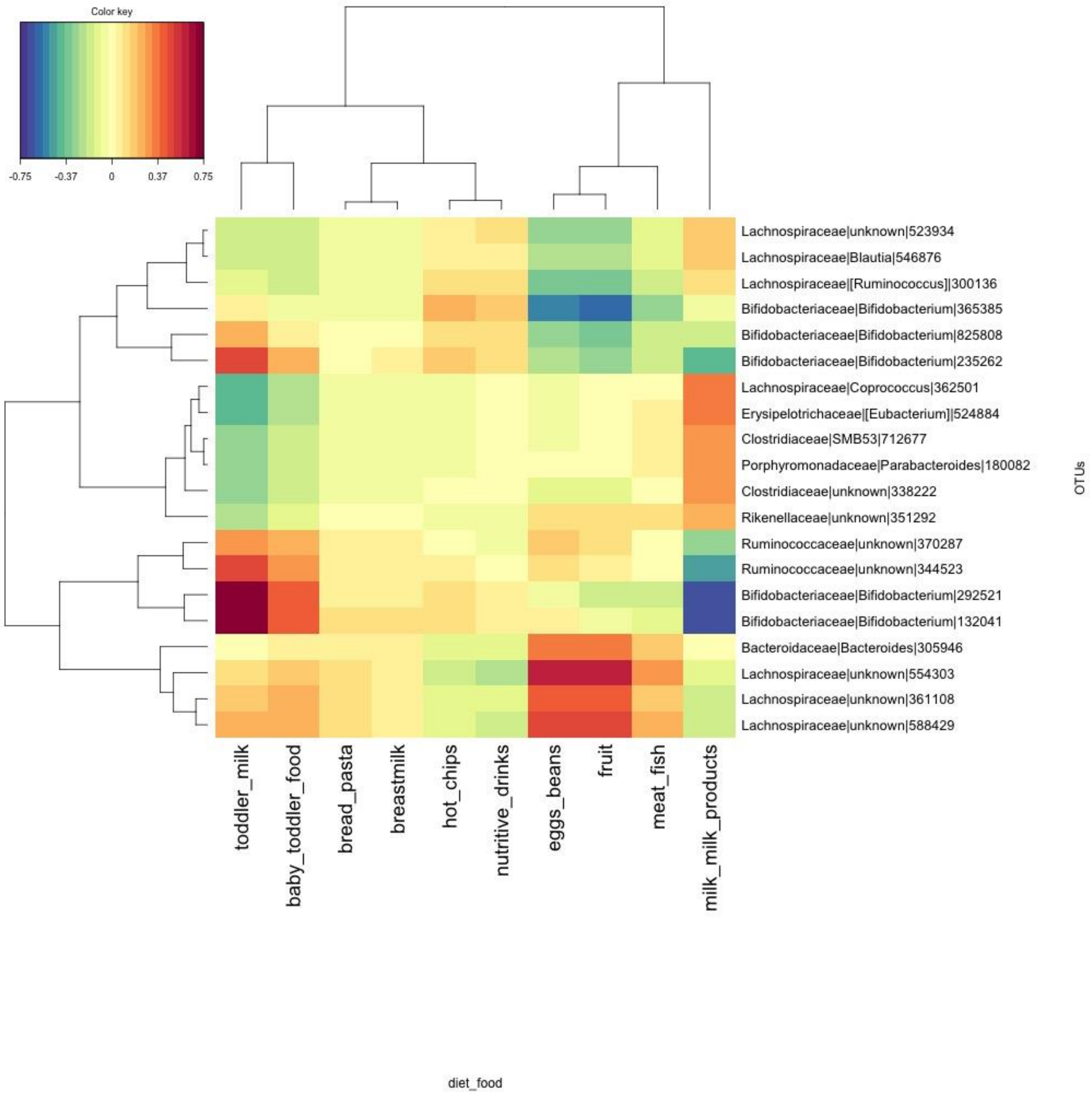
Color key
-0.75
-0.37
0
0.37
0.75
Lachnospiraceae|unknown|523934
Lachnospiraceae|Blautia|546876
Lachnospiraceae|[Ruminococcus]|300136
Bifidobacteriaceae|Bifidobacterium|365385
Bifidobacteriaceae|Bifidobacterium|825808
Bifidobacteriaceae|Bifidobacterium|235262
Lachnospiraceae|Coprococcus|362501
Erysipelotrichaceae|[Eubacterium]|524884
Clostridiaceae|SMB53|712677
Porphyromonadaceae|Parabacteroides|180082
Clostridiaceae|unknown|338222
Rikenellaceae|unknown|351292
Ruminococcaceae|unknown|370287
Ruminococcaceae|unknown|344523
Bifidobacteriaceae|Bifidobacterium|292521
Bifidobacteriaceae|Bifidobacterium|132041
Bacteroidaceae|Bacteroides|305946
Lachnospiraceae|unknown|554303
Lachnospiraceae|unknown|361108
Lachnospiraceae|unknown|588429
OTUs
toddler_milk
baby_toddler_food
bread_pasta
breastmilk
hot_chips
nutritive_drinks
eggs_beans
fruit
meat_fish
milk_milk_products
diet_food

Time point 12 (end of study)

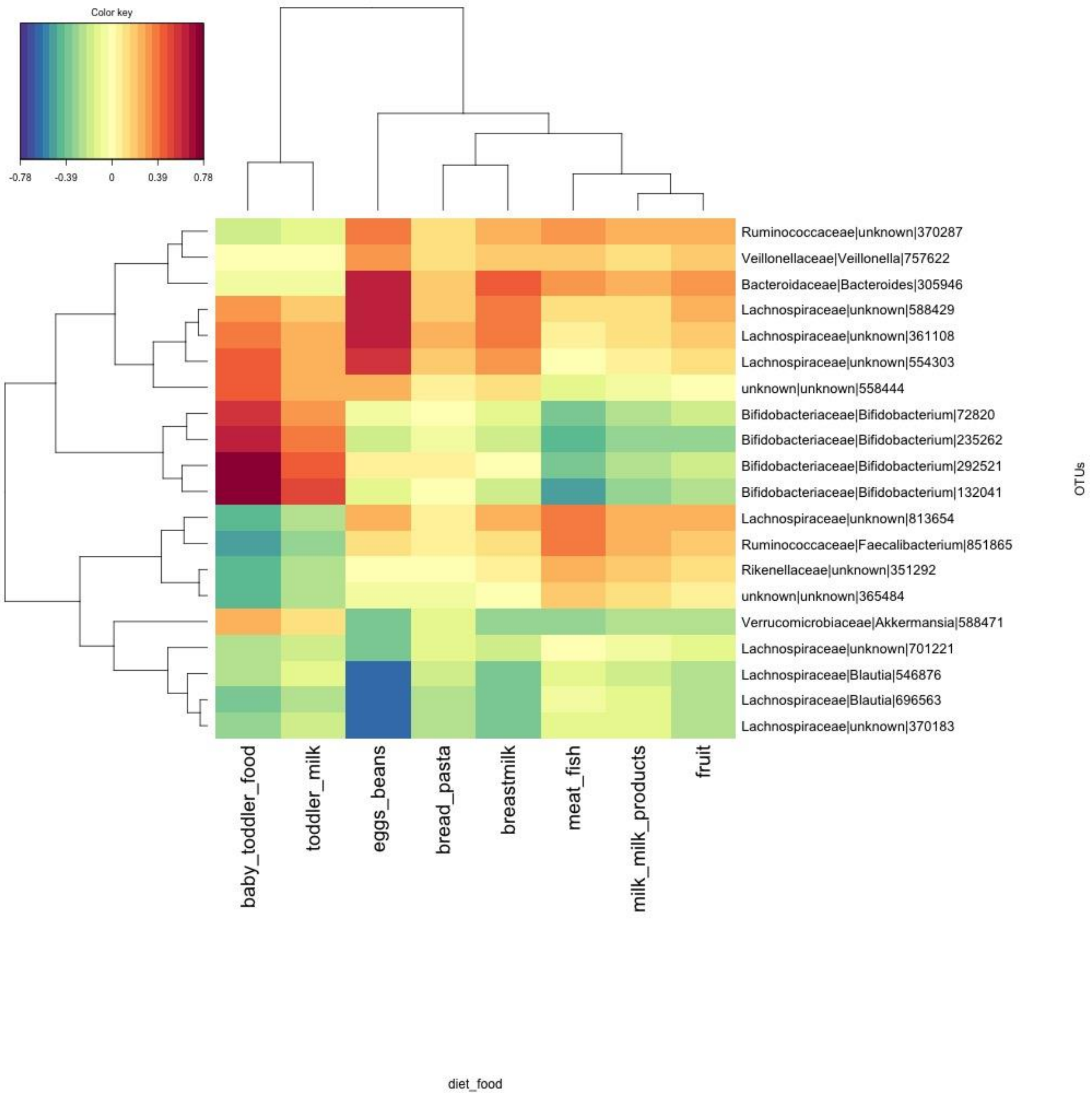

Supplementary Information Details of methodology

Growing Up Milk-Lite (GUMLi) trial and dietary analysis

The Growing Up Milk-Lite (GUMLi) trial participants were randomised to either intervention or control group. The intervention group received GUMLi and the control group received whole pasteurised and homogenised cow's milk, both in powdered form. GUMLi was a standard cow's milk based GUM product that is similar to a commercial product currently on the market, but with a reduced energy and protein content compared to other growing up milk (GUM) in the market. GUMLi was fortified with micronutrients (including vitamin D and iron), probiotic *B. breve* M-16V and prebiotics, long-chain GOS and short-chain FOS. Adherence was checked each month in a monthly questionnaire. Sample size calculation for the GUMLi trial was based on the trial's primary outcome of change in percentage body fat. A sample size of 64 in each of the intervention and control group was required to detect 0.5 of a SD of difference in percentage body fat between each arm. Eighty children were enrolled in each group to allow for an attrition rate of 20%.

GUMLi trial participants in Brisbane were invited to take part in the CHaRM study, with additional consent. Stool samples were collected from one-year old children by their mother or caregiver in a 25 ml faeces container (Sarstedt). The baseline sample was collected prior to starting the GUMLi trial, before children were randomised to trial milk groups. Subsequent stool samples were collected at 1, 2, 3, 6, 9 and 12 months into the trial. Samples were to be collected within ± 2 weeks of due date. Stool samples were immediately stored at -20 ºC prior to transfer to -80 ºC and all samples were transported on ice. On average, samples were taken to -80 ºC storage in 1.57 days. Parents and caregivers were requested to complete the Amsterdam stool chart[1] to indicate the bowel movement of the child up to two days prior to sampling, to identify any abnormalities in the sample collected. Each month, the general bowel habit of the child was asked as part of the monthly questionnaire. The mother or caretaker of the child were asked to withhold taking samples if the child was using antibiotics. These samples were collected seven days post antibiotic cessation, therefore, in some cases samples were collected outside of the due date.

Dietary intake was assessed using the Eating Assessment in Toddlers Food Frequency Questionnaire (EAT FFQ)[2], a previously validated interviewer-administered FFQ specifically developed for children aged 12 to 24 months. The EAT FFQ was designed to assess dietary habit of young children over the previous four weeks and rank children by nutrient intake and dietary pattern score. The EAT FFQ contains 91 food items with questions split into 11 categories. The common foods were grouped into 16 food groups (**Table S3**). The frequency of consumption of each food group was used for dietary pattern analysis. The FFQ data were obtained at 0, 3, 6, 9, and 12 months of the study.

QIIME workflow

We used 'fastq-join' command to join paired-ends. This was followed by splitting the libraries using the following scripts:

```
(cd joined_output && ls –d */) > output_names.
```

```
sed 's_/_/fastqjoin.join.fastq_g' output_names | tr "\n" , | sed 's/,$//' | sed
's/^/split_libraries_fastq.py -i /' > step1
```

```
tr "\n" , < sample_ids.txt | sed 's/,$/ --barcode_type 'not-barcoded' -q 19 -o split -m dummy_map.txt/' | sed 's/^/ --sample_id /' > step2

cat step1 step2 > step3

sed -i "1 i\shellscript" step3

sed 's/shellscript/#!\/bin\/sh\n/' step3 > run_split_script.sh

chmod 700 run_split_script.sh

./run_split_script.sh
```

The OTUs were then assigned using pick_open_refrence_otus.py command.

Supplementary Information Reference List

1 Bekkali, N., Hamers, S. L., Reitsma, J. B., Van Toledo, L. & Benninga, M. A. Infant stool form scale: development and results. *J Pediatr* **154**, 521-526 e521, doi:10.1016/j.jpeds.2008.10.010 (2009).

2 Mills, V. C. *et al.* Relative validity and reproducibility of a food frequency questionnaire for identifying the dietary patterns of toddlers in New Zealand. *J Acad Nutr Diet* **115**, 551-558, doi:10.1016/j.jand.2014.09.016 (2015).
